# Treatment at an Academic Medical Center Eliminates Survival Disparities for Appalachian Kentuckians with Pancreatic Ductal Adenocarcinoma

**DOI:** 10.13023/jah.0601.02

**Published:** 2024-09-01

**Authors:** Emily Cassim, Hannah McDonald, Megan Harper, Quan Chen, Miranda Lin, Reema Patel, Michael Cavnar, Prakash Pandalai, Bin Huang, Pamela C. Hull, Joseph Kim, Erin Burke

**Affiliations:** University of Kentucky; The University of Kentucky; University of Kentucky; University of Kentucky

**Keywords:** Appalachia, pancreatic ductal adenocarcinoma (PDAC), pancreatic cancer, healthcare disparities, Kentucky, treatment outcomes

## Abstract

**Introduction:**

Rates of cancer mortality in Appalachian Kentucky is among the highest in the nation. It is unknown whether geographic location of treatment for pancreatic ductal adenocarcinoma (PDAC), one of the deadliest cancers worldwide, influences survival in Appalachian Kentuckians.

**Purpose:**

This study compares outcomes among Appalachian Kentuckians with PDAC who received treatment at an academic medical center (AMC) or community facility (CF).

**Methods:**

Using the Kentucky Cancer Registry, patients diagnosed with PDAC between 2003 and 2018 were identified. Patients were categorized according to treatment location (AMC v. CF) and county of residence (Appalachian v. non-Appalachian). Kaplan-Meier curves were constructed to assess survival and multivariate Cox regression analyses were performed.

**Results:**

Overall, out of 4,402 PDAC patients, 87.3% received treatment at CFs and 12.7% at an AMC. When stratified by treatment location and Appalachian status, significant differences were found in clinicopathologic factors, such as age, smoking, insurance status, stage, and treatment (*p* < .05). Factors significantly associated with decreased survival included treatment at a CF (HR 1.53 for Appalachian, 1.25 for Non-Appalachian), patient age > 75 years (HR 1.44), having Medicare/Medicaid insurance (HR 1.23/1.16), and history of smoking (HR 1.11). Decreased 1- and 5-year survival was associated with treatment at a CF for both Appalachian (27.4% and 3.6%) and Non-Appalachian (36% and 5.7%) patients (*p* < .001).

**Implications:**

Improved survival of Kentuckians treated at an AMC suggests that poorer PDAC outcomes in Appalachian patients may be related to access to tertiary care. Future research should examine potential reasons for these disparate outcomes and strategies for increasing the quality of cancer care at CFs.

## INTRODUCTION

The Appalachian Region has 26 million residents spanning 13 states from southern New York to northern Mississippi. Nearly half of the counties in the Commonwealth of Kentucky are in Appalachia. Unfortunately, Kentucky has the greatest medical and economic distress within the Region and mortality rates from cancer, heart disease, chronic obstructive pulmonary disease, and traumatic injury in Appalachian Kentucky far exceed those in the rest of Appalachia and the wider U.S.^1^,^2^ In fact, cancer-related mortality in Appalachian Kentucky is 227 per 100,000 persons, which is 35% higher than the national average.^2^ Pancreatic ductal adenocarcinoma (PDAC) will soon be the second-leading cause of cancer-related deaths, and Kentuckians historically have higher PDAC mortality rates than national averages (13 and 9.8 v. 12.7 and 9.6 for male and female patients, respectively).^3^–^5^

The etiology for poorer outcomes in Appalachian Kentucky is multifactorial, including socioeconomic disparities and barriers to healthcare access. Barriers to access in this region are often due to the inability to miss work, hire childcare or travel more than 3 hours to reach the nearest academic medical centers (AMCs). Such obstacles are difficult to overcome for most Appalachian Kentuckians, who have high rates of household poverty (26.7%) and disability (17.3%) and low education levels (21% < high school education).^2^ Additionally, this region has a shortage of primary care physicians and specialists. Indeed, the number of primary care physicians in Appalachian Kentucky is 36% lower than the national average and 20% lower than the rest of the Appalachian Region.^2^ Specialists, such as oncologists, are also in short supply, with only 62 oncologists per 100,000 people in Appalachia. In contrast, non-Appalachian Kentucky has 156 oncologists per 100,000 people, which is 86% higher than in the Appalachian Region and roughly equivalent to the national average of 153 oncologists per 100,000 people.^2^ These data show that while there is a sufficient number of physicians to provide medical care in Kentucky as a whole, the Appalachian portion of Kentucky continues to face considerable physician shortages.

Specific factors contributing to survival disparity remain uncertain. The aforementioned socioeconomic disparities and barriers to healthcare access may explain why Appalachian Kentuckians have poor PDAC survival. However, other reports have shown the importance of the volume and quality of care in relation to treatment outcomes.^6^ This study aims to determine whether treatment setting contributes to the survival disparity observed in Appalachian Kentuckian patients with PDAC. To assess this, treatment and survival outcomes of Kentucky PDAC patients who were treated at an AMC were compared to patients treated at community facilities (CFs). It was hypothesized that when Appalachian Kentuckians received treatment at an AMC, PDAC survival disparities would be reduced or eliminated.

## METHODS

### Patient Population

Data analysis was performed using the Kentucky Cancer Registry (KCR) and included patients diagnosed with pancreatic cancer in Kentucky from 2003 to 2018. Of 8,545 total patients with pancreatic cancer, 7,823 with histology codes corresponding to invasive PDAC (ICD-O-3 histology codes 8000–8004, 8010, 8012, 8020, 8050, 8140, 8141, 8144, 8230, 8255–8257, 8260–8263, 8290, 8440–8443, 8450–8454, 8460–8463, 8470–8474, 8480, 8481, 8500, 8503, 8504, 8521, 8560, and 8570) were identified. A total of 3,421 patients who had no available data for treatment type or treatment location were then excluded, leaving a total of 4,402 patients.

### Treatment Location

Patients were analyzed and compared based on residence and treatment location: AMC (*i.e*., the University of Kentucky, UK; and its NCI-designated Comprehensive Cancer Center) v. CFs and Appalachian (AP) v. Non-Appalachian (Non-AP) patients. Patients in the AMC treatment group received some or all of their treatment at UK. Patients in the CF treatment group received all of their treatment at non-UK locations. To compare Appalachian to non-Appalachian individuals, patients were categorized by ZIP code of residence. Appalachian patients were defined as residing within the 54 Kentucky Appalachian counties as defined by the Appalachia Regional Commission.^1^ To further understand the relationship and interaction between residence and treatment location, patients were classified into four groups: AP patients at AMC, AP patients at CF, Non-AP patients at AMC, and Non-AP patients CF.

### Stage and Treatment Modality

Stage of disease and treatment were defined based on the KCR’s 2021 Abstractor’s Manual. Stage was defined as localized, regional, or distant. Treatments were divided into surgery, radiation, chemotherapy, and other as the first course treatment. Surgery included both curative intent and palliative intent. KCR defines “other” as non-definitive treatments that are not tumor-directed but prolong a patient’s life, make them comfortable, or prepare the patient for definitive treatment, such as chemotherapy, radiation, or surgery.

### Patient Characteristics

Within the PDAC cohort, age was divided into the following groups: 20–49 years, 50–64 years, 65–74 years, and ≥ 75 years at the time of diagnosis. Race was defined as white, black, or other. Insurance status was categorized as uninsured, private insurance, Medicaid, Medicare, or unknown. Positive smoking status was defined as any tobacco use during the patient’s lifetime.

Descriptive analyses were performed to evaluate age, gender, race, insurance status, Appalachian residence, smoking status, and PDAC treatment locations using χ^2^ tests. Kaplan-Meier survival curves were constructed to determine 1- and 5-year survival according to stage at diagnosis, treatments received, Appalachian residence, race, and treatment location. Cox regression analysis was utilized to identify factors that were independently associated with survival.

### Statistical Analysis

Patients’ demographic and clinical counts and percentages were summarized by Appalachian residence and treatment location. The inter-group difference was compared using χ^2^ tests. Kaplan-Meier plots were examined to compare survival curves among the combination of treatment location and Appalachian residence status, and *p*-values based on log-rank tests were provided. Cox regression analysis was utilized to identify factors that were independently associated with survival. Two-way interaction effects were examined, and significant interaction effects were included in the final Cox regression model. All statistical tests were two-sided, with a significance level of *.05*. All analyses were performed utilizing SAS/STAT version 9.4 (Cary NC).

## RESULTS

### Patient Population

After excluding patients with unknown treatment location and types of treatment, a total of 4,402 patients were analyzed. Most patients (54.2%) were aged 65 years or older (Table 1). Male and female sex were nearly equivalent. In relation to race, 92.1% of patients were white, 7.4% were black, and 0.5% were another or unknown race. These numbers for PDAC patients were similar to the overall demographics of the Commonwealth of Kentucky’s population, which is 87.5% white and 8.5% black.^7^ Over one-half (51.9%) of patients smoked, whereas 31.6% did not smoke; smoking status was unknown in 16.5%. Medicare was the most common form of health insurance, followed by private insurance. When assessing distribution of treatment location, 558 (12.7%) received treatment at an AMC and 3,844 (87.3%) patients were treated exclusively at CFs. Regarding Appalachian status, 3,261 (74.1%) of patients were non-AP and 1,141 (25.9%) were AP.

### Demographics by Treatment Location OR Appalachian Status

Regarding the demographics of AMC and CF patients, respectively: 56.8% and 21.4% were AP; 48.2% and 52.8% were male; 94.1% and 91.8% were white; and 5.6% and 7.7% were black. The percentage of AMC patients who had Medicaid was lower (48.2% v. 57.5%, *p <* .0001), who had private insurance was higher (31% v. 23.3%, *p <* .0001), and who had Medicare was similar (16.1% v. 15.8%, *p <* 0.0001) to percentages for CF patients. AMC and CF patients also had grossly similar rates of smoking (55.4% v. 51.4%) (see Supplemental Table 1 [SuppTbl 1] in the Additional Files section online). Pairwise comparison of non-AP and AP demographics showed similar age ≥ 65 years (55.1% and 51.4%), sex (49.7% and 54.5% male, *p =* 0.0723), smoking status (51.6% and 52.6%, *p =* 0.7194) and insurance status. However, there was a difference in race (9.6% and 1.1% black; 89.7% and 98.9% white, *p <* 0.0001) (SuppTbl 1).

### Demographics by Treatment location AND Appalachian Status

Familywise comparison of AP patients at AMC, AP patients at CF, Non-AP patients at AMC, and Non-AP patients at CF revealed significant differences in age, gender, race, smoking, and insurance status (Table 1). A higher proportion of patients under age 65 years were treated at an AMC (55.2% AP and 54.3% Non-AP), with a higher proportion of older patients (65+) being treated at a CF (53.9% AP and 55.8% Non-AP). There were more Non-AP female patients at AMCs (54.4%) and fewer at AP female patients at CFs (43.8%). There was a higher proportion of white AP patients at both AMC (98.4%) and CF (99%). Interestingly, smoking was more prevalent in Non-AP patients at AMCs (58.1%); however, when considering only patients whose smoking status was known, there was a higher rate of smoking in AP patients treated at CFs (63.3%). Non-AP patients treated at an AMC were more likely to have private insurance (34%); AP patients treated at a CF were more likely to have Medicare (60.4%) and Medicaid (20.6%).

### PDAC Stage and Treatment

For Kentucky PDAC patients, stage at diagnosis was 9% localized, 41.8% regional, 47.0% distant, and 2.2% unknown. These values differ from disease stages at diagnosis for the U.S. population, which were 12.3% localized, 28.4% regional, 48.3% distant, and 11% unknown.^5^ When analyzing treatment modalities for all patients—many of whom received multiple treatment types—31.0% received surgery, 85.6% received chemotherapy, 29.8% received radiation, and 2.8% received “other” treatment.

### Stage and Treatment by Location OR Appalachian Status

In Non-AP patients v. AP patients, disease stage at diagnosis was similar: 9.0% v. 9.3% localized; 41.7% v. 41.8% regional; 47.2% v. 46.3% distant; and 2.2% v. 8.9% unknown, respectively (SuppTbl 1). However, patients treated at AMCs v. CFs differed significantly by stage of disease. Those treated at an AMC v. a CF had more localized (10.4% v. 8.8%) and regional (52.3% v. 40.2%) disease, and fewer patients with distant metastases (35.7% v. 48.6%); a similar percentage (2.8% v. 2.4%) had unknown stage of disease. Patients treated at an AMC more often received surgery (37.8% v. 30%, *p =* .0002) and radiation therapy (44.1% v. 27.7%, *p* < .0001) than those treated at a CF (SuppTbl 1). Treatment types for patients at an AMC v. a CF were assessed when stratified by disease extent. Patients with localized disease received similar rates of surgery (46.6% v. 41.5%, *p* = .48), radiation (39.7% v. 37.4%, *p* = .74), and chemotherapy (74.1% v. 69.7%, *p =* .5), respectively. Patients with regional disease received similar rates of surgery (55.1% v. 55.3%, *p =* .96), but more radiation (53.4% v. 43.6%, *p =* .002) and chemotherapy (86.7% v. 79.4%, *p =* .0043) at the AMC. Patients with metastatic disease received similar rates of surgery (10.1% v. 7.1%, *p =* .13), more radiation (30.2% v. 12.2%, *p <* .0001), and less chemotherapy (83.9% v. 94.1%, *p* < .0001) at the AMC (SuppTbl 1).

### Stage and Treatment by Location AND Appalachian Status

Familywise comparison of AP patients at AMC, AP patients at CF, Non-AP patients at AMC, and non-AP patients at CF revealed significant differences in stage and treatment (surgery, radiation, and other) (Table 1). Appalachian patients treated at an AMC had higher rates of locoregional disease, while Appalachian patients treated at a CF had higher rates of distant or metastatic disease. Furthermore, Appalachians treated at a CF were less likely to receive surgery or radiation, with no difference in receipt of chemotherapy.

### Survival Analysis

With Cox regression analysis, significant factors and interaction effects were included in the final model. Several factors were associated with overall survival. Both AP (HR 1.31, *p <* .001*)* and Non-AP (HR 1.17, *p =* .033) patients treated at a CF had decreased survival compared to those treated at an AMC (Table 2). Interestingly, black patients had higher survival rates (HR 0.85, *p =* .008), patients aged 75 years and older had poorer survival (HR 1.33, *p <* .001), as did those with Medicaid (HR 1.12, *p =* .034) and Medicare (HR 1.12, *p =* .032) insurance. Those who had a history of smoking had decreased survival (HR 1.13, *p <* .001) as well. Surgery (HR 0.4, *p <* .001) and radiation (HR 0.7, *p <* .001) were associated with improved survival, whereas chemotherapy administration trended towards decreased survival (HR 1.1, *p =* .06). More advanced stage, as would be expected, was associated with poorer survival, as well (Table 2). Significant interactions were found between stage and radiation (*p* = .041), as well as stage and chemotherapy (*p* < .001).

**Figure 1:**
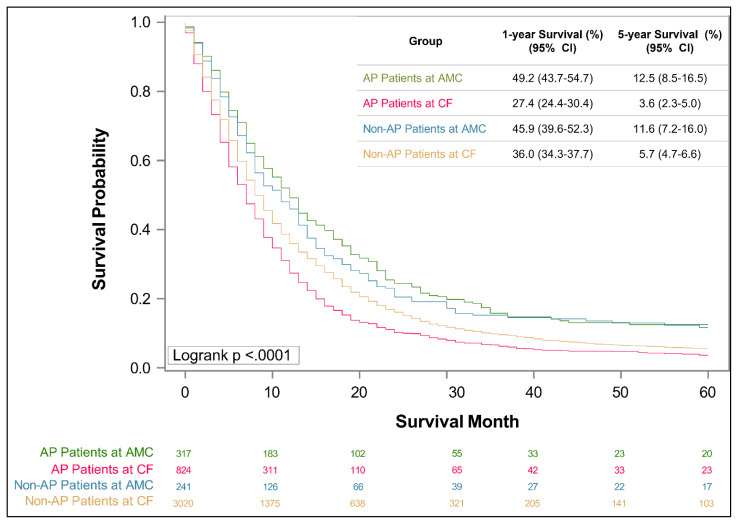
Kaplan Meier Curve for 1- and 5-year Survival of Four Groups Stratified by Treatment Location and Appalachian Status

According to KCR data, Kentuckians have a 5-year PDAC survival rate of 16% for localized disease, 8% for regional disease, and 0.9% for distant disease. In comparison of 1- and 5-year survival rates, stratified by both treatment location and Appalachian status, 1- and 5-year survival was significantly lower for patients treated at a CF (Fig. 1, *p* < .0001). For AP patients at a CF, 1- and 5-year survival was the lowest, with rates of 27.4% and 3.6%, respectively. For Non-AP patients at CF, 1- and 5-year survival was 36% and 5.7%, respectively. AP and Non-AP patients had similar survival when treated at an AMC, however—with 1- and 5-year rates of 49.2% and 12.5% (AP patients), and of 45.9% and 11.6% (Non-AP patients).

## DISCUSSION & IMPLICATIONS

Historically, Kentucky has had poorer survival rates for all cancer types compared to the rest of the nation.^3^–^5^ It is a great public health concern that the Appalachian region of Kentucky has survival outcomes that are even lower than Kentucky state averages.^2^ This analysis of Kentucky patients diagnosed with PDAC between 2003 and 2018 highlights survival disparities for Kentucky PDAC patients, as well as more severe disparities for Appalachian Kentuckians. The data show that treatment at an AMC appears to overcome Appalachian disparities by improving survival to the rates observed at national levels. These improved outcomes highlight the potential to eliminate healthcare disparities by increasing access to AMCs.^8^,^9^

The study results exhibiting improved survival at an AMC are consistent with prior reports.^10^–^12^ Indeed, these findings suggest the existence of treatment-related disparities between AMCs and CFs. One explanation for the improved survival at AMCs may be their increased number of resources. AMCs often have access to newer healthcare technology and provide patients with access to clinical trials, as they are centers for education and research. AMCs also receive more government funding, often directly impacting the condition of their facilities. This is important, as it has been shown that the condition of a hospital’s facilities directly influences quality of care and perioperative mortality.^13^ Although CFs and AMCs had similar rates of surgery for localized, regional, and distant disease, outcomes for CF patients were worse.

This observed worse survival is likely multifactorial, however it could be related to the shortage of experienced oncologists who practice at CFs. Outcomes are routinely better at AMCs, which have a larger number of surgical specialists with expertise to perform complex cancer surgery.^12^,^14^ AMCs often have higher patient and surgery volume than CFs, which have been consistently correlated with improved outcomes.^15^,^16^ PDAC is also managed by a multidisciplinary approach with medical, surgical, and radiation oncologists, as well as pathologists, radiologists, and other subspecialized practitioners.^17^,^18^ This multidisciplinary management is associated with improved survival, and a comprehensive group of specialists are less likely at smaller CFs.^17^,^18^ AMCs, such as UK, are also more likely to be NCI-designated centers for excellence in cancer treatment, which are shown to have improved outcomes.^19^

Interestingly, this study also found that in Kentucky, 5-year survival was higher for black patients than white patients. It also showed that black patients had higher survival when treated at CFs. This is inconsistent with current literature, where black patients have decreased 5-year PDAC survival compared to white patients.^20^ We hypothesize that this incongruency may be secondary to the relatively smaller numbers of black patients in the study cohort. We also acknowledge the potential limitation of the retrospective nature of reviewing patient data from a population-based cancer registry (SEER) and the KCR. There may be underreported or incomplete data, variations in data reporting, coding errors, migration of patients in and out of SEER registry areas, and selection bias. Although 7,823 Kentucky patients with PDAC were identified between 2003 and 2018, treatment data were only available for 4,402 of them. This may introduce bias, as it was only possible to calculate survival rates for patients whose treatment data were available. It was not possible to assess here other factors which may impact survival outcomes, including patient education level, income, compliance to treatment, patient choice, and details regarding treatment regimens, as these data points were not collected in this registry.

Despite the above limitations, the study results show that improved survival was observed in patients with PDAC who received care at an AMC. Importantly, these findings highlight an opportunity to improve PDAC survival for patients in Appalachian Kentucky.

**SUMMARY BOX**

**What is already known about this topic?**

Previous research has shown that there is a lower rate of cancer survival for Kentuckians, particularly from the Appalachian region. However, the impact that treatment location has on survival for Kentucky Appalachian patients was unknown.

**What is added by this report?**

This study found that treatment at an AMC was associated with improved survival for all patient populations. Our findings demonstrated that location of treatment impacts survival outcomes in PDAC patients, especially those from the Appalachian region of Kentucky. This promising data showed that treatment at an AMC may improve outcomes in patient populations with healthcare disparities such as low socioeconomic status or geographic barriers to care.

**What are the implications for future research?**

Findings show that AMCs are invaluable resources for Kentucky patients.

## Supplementary Information



## Figures and Tables

**Table 1:** Familywise Comparison of Clinicopathologic Data by Treatment Location and Appalachian Status

Characteristic	Category	Total (N = 4402)	AP Patients at AMC (n = 317)	AP Patients at CF (n = 824)	Non-AP Patients at AMC (n = 241)	Non-AP Patients at CF (n = 3020)	*p*-value (two sided)
Age Group (yrs)	20–49	333(7.6)	36(11.4)	65(7.9)	23(9.5)	209(6.9)	< .0001

	50–64	1685(38.3)	139(43.8)	315(38.2)	108(44.8)	1123(37.2)	

	65–74	1472(33.4)	98(30.9)	289(35.1)	87(36.1)	998(33)	

	75+	912(20.7)	44(13.9)	155(18.8)	23(9.5)	690(22.8)	

Gender	Male	2299(52.2)	159(50.2)	463(56.2)	110(45.6)	1567(51.9)	.0181

	Female	2103(47.8)	158(49.8)	361(43.8)	131(54.4)	1453(48.1)	

Race	White	4054(92.1)	312(98.4)	816(99)	213(88.4)	2713(89.8)	< .0001

	Black	326(7.4)	4(1.3)	8(1)	27(11.2)	287(9.5)	

	Other/Unknown	22(0.5)	1(0.3)	0(0)	1(0.4)	20(0.7)	

Smoking	No	1391(31.6)	109(34.4)	250(30.3)	77(32)	955(31.6)	.0235

	Yes	2283(51.9)	169(53.3)	431(52.3)	140(58.1)	1543(51.1)	

	Unknown	728(16.5)	39(12.3)	143(17.4)	24(10)	522(17.3)	

Insurance	Not insured	126(2.9)	10(3.2)	21(2.5)	8(3.3)	87(2.9)	< .0001

	Private insured	1068(24.3)	91(28.7)	126(15.3)	82(34)	769(25.5)	

	Medicaid	698(15.9)	46(14.5)	170(20.6)	44(18.3)	438(14.5)	

	Medicare	2479(56.3)	165(52.1)	498(60.4)	104(43.2)	1712(56.7)	
	Unknown	31(0.7)	5(1.6)	9(1.1)	3(1.2)	14(0.5)	

Stage	Localized	395(9.0)	41(12.9)	65(7.9)	17(7.1)	272(9)	< .0001

	Regional	1838(41.8)	170(53.6)	307(37.3)	122(50.6)	1239(41)	

	Distant	2067(47.0)	100(31.5)	428(51.9)	99(41.1)	1440(47.7)	

	Unknown	102(2.2)	6(1.9)	24(2.9)	3(1.2)	69(2.3)	

Surgery	No	3038(69.0)	189(59.6)	628(76.2)	158(65.6)	2063(68.3)	< .0001

	Yes	1364(31.0)	128(40.4)	196(23.8)	83(34.4)	957(31.7)	

Radiation	No	3092(70.2)	175(55.2)	609(73.9)	137(56.8)	2171(71.9)	< .0001

	Yes	1310(29.8)	142(44.8)	215(26.1)	104(43.2)	849(28.1)	

Chemotherapy	No	636(14.4)	57(18)	119(14.4)	30(12.4)	430(14.2)	.2521

	Yes	3766(85.6)	260(82)	705(85.6)	211(87.6)	2590(85.8)	

Other Treatment	No	4278(97.2)	281(88.6)	804(97.6)	229(95)	2964(98.1)	< .0001

	Yes	124(2.8)	36(11.4)	20(2.4)	12(5)	56(1.9)	

NOTES:

* CF=community facility, AMC = Academic medical center, Non-AP = Non-Appalachian, AP = Appalachian

**Table 2:** Estimates of Univariate and Multivariable Cox Regression Models

Factors	Univariate Hazard Ratio (95% CI)	*p*-value	Multivariable Hazard Ratio (95% CI)	*p*-value
**Appalachian Residence and Therapy Location**				
Non-AP Patients at AMC	1.0		1.0	
AP Patients at AMC	0.92 (0.77–1.11)	.390	0.99 (0.82–1.18)	.887
AP Patients at CF	1.53 (1.31–1.78)	< .001	1.31 (1.12–1.53)	< .001
Non-AP Patients at CF	1.25 (1.09–1.44)	.002	1.17 (1.01–1.35)	.033
**Race**				
White	1.0		1.0	
Black	0.84 (0.75–0.95)	.003	0.85 (0.75–0.96)	.008
**Age**				
20–49	1.0		1.0	
50–64	1.12 (0.99–1.27)	.073	1.09 (0.96–1.29)	.193
65–74	1.18 (1.04–1.33)	.012	1.12 (0.96–1.29)	.145
> 75 years	1.44 (1.26–1.64)	< .001	1.33 (1.14–1.56)	< .001
**Insurance**				
Private	1.0		1.0	
Not insured	1.15 (0.95–1.40)	.154	1.18 (0.97–1.44)	.102
Medicaid, other	1.16 (1.05–1.28)	.004	1.12 (1.01–1.24)	.034
Medicare	1.23 (1.14–1.32)	< .001	1.12 (1.01–1.24)	.032
Unknown	1.56 (1.08–2.24)	.0167	1.31 (0.91–1.89)	.147
**Smoking**				
No	1.0		1.0	
Yes	1.11 (1.03–1.19)	.0125	1.13 (1.05–1.22)	< .001
**Surgery**				
No	1.0		1.0	
Yes	0.36 (0.34–0.39)	< .001	0.40 (0.37–0.44)	< .001
**Radiation** †				
No	1.0			
Yes	0.70 (0.65–0.74)	< .001		
**Chemotherapy** †				
No	1.0			
Yes	1.1 (1.0–1.19)	.06		
**Stage** †				
Localized	1.0			
Regional	1.21 (1.07–1.37)	.002		
Distant	2.64 (2.34–2.98)	< .001		
Unknown	1.39 (1.11–1.76)	.005		

NOTES:

* CF=community facility, AMC = Academic medical center, Non-AP = Non-Appalachian, AP = Appalachian

† The following significant interactions were included in the multivariable Cox regression: radiation therapy and stage (*p* = .041); chemotherapy and stage (*p* < .001).
