# Supplementary material for: Treatment at an Academic Medical Center Eliminates Survival Disparities for Appalachian Kentuckians with Pancreatic Ductal Adenocarcinoma
Source: J Appalach Health. 2024 Sep 1;6(1-2):6–20. doi: 10.13023/jah.0601.02 (PMC11617027; doi:10.13023/jah.0601.02)
Supplement: Supplementary file 1 [file jah-6-1-2-6-Additionalfile.docx]

| **Supplementary Table 1.** Pairwise Comparison of Patient Characteristics by Treatment Location and County of Residence  *CF=community facility, AMC = Academic medical center, Non-AP = Non-Appalachian, AP = Appalachian | | | | | | | |
| --- | --- | --- | --- | --- | --- | --- | --- |
|  | **Total** | **CF** | **AMC** |  | **Non-AP** | **AP** |  |
| **Factors** | n = 4402 (n %) | 3844 (87) | 558 (12.7) | ***P*** | 3261 (74.1) | 1141 (25.9) | ***P*** |
| **Age** |  |  |  |  |  |  |  |
| 20-49 | 333 (7.6) | 274 (7.1) | 59 (10.6) | <0.0001 | 232 (7.1) | 101 (8.9) | 0.0062 |
| 50-64 | 1685 (38.3) | 1428 (37.4) | 247 (44.3) |  | 1231 (37.7) | 454 (39.8) |  |
| 65-74 | 1472 (33.4) | 1287 (33.5) | 185 (33.2) |  | 1085 (33.3) | 387 (33.9) |  |
| 75+ | 912 (20.7) | 845 (22.0) | 67 (12.0%) |  | 713 (21.9) | 199 (17.4) |  |
| **Gender** |  |  |  | 0.042 |  |  |  |
| Male | 2299 (52.2) | 2030 (52.8) | 269 (48.2) |  | 1677 (51.4) | 622 (54.5) | 0.0723 |
| Female | 2103 (47.8) | 1814 (47.2) | 289 (51.8) |  | 1584 (48.6) | 519 (45.5) |  |
| **Race** |  |  |  | 0.1751 |  |  |  |
| White | 4054 (92.1) | 3528 (91.8) | 525 (94.1) |  | 2926 (89.7) | 1128 (98.9) | <0.0001 |
| Black  Other | 326 (7.4) | 295 (7.7)  20 (0.5%) | 31 (5.6%)  2 (0.3%) |  | 314 (9.6)  21 (0.7%) | 12 (1.1)  1 (0.09%) |  |
| **Insurance** |  |  |  | <0.0001 |  |  |  |
| Uninsured | 126 (2.9) | 108 (2.8) | 18 (3.2) |  | 95 (2.9) | 31 (2.7) | <0.0001 |
| Private | 1068 (24.3) | 895 (23.3) | 173 (31.0) |  | 851 (26.1) | 217 (19.0) |  |
| Medicare | 698 (15.9) | 608 (15.8) | 90 (16.1) |  | 482 (14.8) | 216 (18.9) |  |
| Medicaid, other | 2479 (56.3) | 2210 (57.5) | 269 (48.2) |  | 1816 (55.7) | 663 (58.1) |  |
| Unknown | 31 (0.7) | 23 (0.60) | 8 (1.4) |  | 17 (0.5) | 14 (1.2) |  |
| **Smoking** |  |  |  | <0.0001 |  |  |  |
| No | 1391 (31.6) | 1205 (31.3) | 186 (33.3) |  | 1032 (31.6) | 359 (31.5) | 0.7194 |
| Yes | 2283 (51.9) | 1974 (51.4) | 309 (55.4) |  | 1683 (51.6) | 600 (52.6) |  |
| Unknown | 728 (16.5) | 665 (17.3) | 63 (11.3) |  | 546 (16.7) | 182 (15.9) |  |
| **Surgery** |  |  |  | 0.0002 |  |  |  |
| No | 3038 (69) | 2691 (70.0) | 347 (62.2) |  | 2221 (68.1) | 817 (71.6) | <0.0001 |
| Yes | 1364 (31) | 1153 (30.0) | 211 (37.8) |  | 1040 (31.9) | 324 (28.4) |  |
| **Radiation** |  |  |  | <0.0001 |  |  |  |
| No | 3092 (70.2) | 2780 (72.3) | 312 (55.9) |  | 2308 (70.8) | 784 (68.7) | 0.1717 |
| Yes | 1310 (29.8) | 1064 (27.7) | 246 (44.1) |  | 953 (29.2) | 357 (31.3) |  |
| **Chemotherapy** |  |  |  | 0.411 |  |  |  |
| No | 636 (14.5) | 549 (14.3) | 87 (15.6) |  | 460 (14.1) | 176 (15.4) | <0.0001 |
| Yes | 3766 (85.6) | 3295 (85.7) | 471 (84.4) |  | 2801 (85.9) | 965 (84.6) |  |
| **Other Treatment** |  |  |  | <0.0001 |  |  |  |
| No | 4278 (97.2) | 3768 (98.0) | 510 (91.4) |  | 3193 (97.9) | 1085 (95.1) | <0.0001 |
| Yes | 124 (2.8) | 76 (2.0) | 48 (8.6) |  | 68 (2.1) | 56 (4.9) |  |
| **Stage** |  |  |  | <0.0001 |  |  |  |
| Localized | 395 (9) | 337 (8.8) | 58 (10.4) |  | 289 (8.9) | 106 (9.3) | 0.0996 |
| Regional | 1838 (41.8) | 1546 (40.2) | 292 (52.3) |  | 1361 (41.7) | 477 (41.8) |  |
| Distant | 2067 (47) | 1868 (48.6) | 199 (35.7) |  | 1539 (47.2) | 528 (46.3) |  |
| Unknown | 102 (2.3) | 93 (2.4) | 9 (1.6) |  | 72 (2.2) | 102 (8.9) |  |
